# Level of satisfaction and associated factors among patients attending outpatient departments of south Wollo health facilities, Ethiopia

**DOI:** 10.1371/journal.pgph.0000761

**Published:** 2022-07-21

**Authors:** Wolde Melese Ayele, Abdurahman Ewunetu, Muluken Genetu Chanie

**Affiliations:** 1 Department of Epidemiology and Biostatistics, School of Public Health, College of Medicine and Health Sciences, Wollo University, Dessie, Ethiopia; 2 Department of Internal Medicine, Dessie Referral Hospital, Dessie City, South Wollo, Ethiopia; 3 Department of Health Systems and Policy, School of Public Health, College of Medicine and Health Sciences, Wollo University, Dessie, Ethiopia; Babcock University, NIGERIA

## Abstract

**Background:**

Patient satisfaction is a key metric for determining how efficient healthcare is delivered. When patients visit health care facilities, they express a clear desire for high-quality services. Inadequately meeting their anticipated needs and expectations may lead to disappointment. This study sought to investigate the level of satisfaction expressed by participants regarding services provided by outpatient departments of selected health facilities in the south Wollo zone of Ethiopia and associated predictors.

**Methods:**

A facility-based cross-sectional study with a total sample of 540 patients was conducted from May 13 to 25, 2019. A multistage sampling technique was used to select participants. Data were collected an interviewer-administered structured validated questionnaire. Data analysis was conducted with SPSS version 20 to identify predictor variables, applying bivariate and multivariate logistic regression analysis to determine variables that most significantly predicted the outcome variable of the level of patient-satisfaction at 5% level of significance and 95% confidence interval.

**Results:**

There were 537 participants in the study consisting of males (50.6%) and females (49.4%). An estimated 35.6% of respondents were between the ages of 28 and 37 years. The proportion of respondents high educational attainment was 179 (33.3%), and 155 (28.9%) of respondents reported having receive free health service of charge.

**Conclusion:**

The study’s results revealed that overall client satisfaction was low. Furthermore, the politeness of health service providers, the convenience of the environment for asking questions, and the availability of all prescription drugs were found to have a significant relationship with level of satisfaction with the health center. Health managers and service providers should come up with creative ways to improve health workers’ caring behavior, protect patients’ privacy, and increase patient satisfaction by making all necessary drugs available.

## Background

Patient satisfaction refers to how satisfied patients are with the service offered in terms of meeting their needs and desires. According to health services researchers report, patients who satisfied were more likely to comply with treatment, keep follow-up appointments, and use health services. While patients who dissatisfied were less likely to comply with treatment, keep follow-up appointments, and use health services [1].

Patient satisfaction is one of the most important aspects of healthcare service-quality, which includes treating patients with respect, respecting their needs, and offering services that meet their needs [2]. Patient satisfaction is considered to be one of the most important factors in a healthcare facility’s success. It is much easier to assess the patient’s satisfaction with the services provided than it is to assess the patient’s satisfaction with the services offered. It is also much easier to assess a patient’s satisfaction with the services offered than it is to assess the quality of medical care they receive [3].

Long wait time during registration, doctor visits after registration, examination room privacy, laboratory procedures and doctor revisiting for evaluation with laboratory results, failure to obtain prescribed drugs and supplies from hospital pharmacies, and insufficient information were all observed problems in studies carried in Out Patient Departments (OPD) of various hospitals in Ethiopia [4, 5].

As the quality of healthcare rises, so does the demand for health services. To achieve high quality, the World Health Organization (WHO) now proposes a "people-centered" approach to health care, in which the patient is treated as a whole person with multiple needs, rather than just a disease condition to be managed. Patients’ satisfaction with the services they receive is one way to quantify health-care quality [6]. The modern patient is more informed and educated, has greater access to information, and has higher expectations of the health-care system. As a result, it is now more important than ever to address service delivery problems in this context [7]. A patient with positive attitudes has a better chance of achieving positive results. Negative patient attitudes and disappointment with health-care services lead to low compliance and, in extreme cases, patients resort to negative word-of-mouth, discouraging others from seeking health-care services from the system [8].

Individuals in Africa did not visit their local primary health care centers even for serious illnesses, according to studies, due to a perception of poor quality of care at these centers [9]. Patient satisfaction is important because it is linked to better compliance with doctors’ instructions, timely care seeking by the patient, and greater understanding and retention of information [10]. One of the indicators of the quality of care is patient satisfaction. Its evaluation will aid in the improvement of health care services and delivery based on patient feedback [9].

Some studies on patient satisfaction have been conducted in Ethiopia, but they have primarily focused on hospital settings. There has been little research on level of patient-satisfaction in primary health care settings, particularly among outpatient attendants at health centers. Thus, this study therefore investigated the level of patient-satisfaction among outpatient attendants in South Wollo, Ethiopia. The finding will have a vital impact on the policymakers, health care providers, especially for providers in primary healthcare settings, and clients to give a personal decision on the approach of service seeking to meet their needs and desire.

## Materials and methods

### Study design and setting

A cross-sectional study was conducted among outpatient department customers at health facilities from May 13 to 25, 2019. The study was carried out in south Wollo, one of Amara National Regional State’s fourteen administrative zones. Dessie city is 400 kilometers from Addis Ababa, the capital of the country and 480 kilometers from Bahirdar, the main city of Amara National Regional State. According to the information obtained from Amhara Regional State, Bureau of Finance and Economic Development (BOFED) the current estimated population is 3,095,457. The zone has a total of 11 primary hospitals, 131 health centers, 523 health posts, 134 private primary clinics (*BOFED report*, *2020*).

### Source and study population

All patients who visited the outpatient departments of the chosen health centers were considered the source population, and all patients who visited the outpatient departments of those ten health centers (from the five districts) during the study period were the study population.

### Study variables

The study’s dependent variable was level of patient-satisfaction. The independent variables were socio-demographic characteristics (age, religion, marital status, level of education, occupation), and outpatient service-related characteristics.

### Inclusion and exclusion criteria

During the study period, patients over the age of 18 who visited health centers were included. Patients who were seriously ill at the time of data collection were excluded because they were not respond properly.

### Sample size determination and sampling procedures

The following assumptions were used to calculate the required sample size using a single population proportion formula. Overall patient satisfaction in health care services is 80.1 percent [5], with an accuracy of 3% at a 95% confidence level and a 10% non-response rate.


$$n=\frac{Z_{1-\alpha /2}^{2}p(1-p)}{d^{2}}=\frac{\left(1.96)2*0.801(1-0.801\right)}{(0.05)2}=245$$

With a 10% non-response rate and a design effect of 2, the final sample size was n = 540. The sample size for the second objective was also computed using Epi-Info version 7 by applying double population proportion formula. The following assumptions: 95% confidence level, 80% power, 3.89 odds ratio, and 10% non-response were used to calculate the sample size for the second objective. However, the sample size was smaller than the sample size obtained for the first objective. Hence, the final sample size of the current study was 540 clients.

As a sampling technique, first multistage sampling technique was used. Five districts in the south Wollo zone were selected by lottery from a total of 24. (Wuchalie, Harbu, Bati, Mekaneselam and Albuko). Two health centers (HC) from each of these districts were selected again by lottery from the available HCs. Finally, the samples were drawn using a simple random sampling method, which involved taking the average daily patient flow from the adult outpatient department of the chosen HCs over the previous year’s same month. Prior to data collection, the total samples were proportionally assigned to the HCs.

### Operational definitions

#### Patient waiting time

The time between receiving service at the next outpatient station and departing from the previous outpatient station.

#### Patient satisfaction

Patients’ perceived needs and expectations in relation to factors such as the health care provider and amenities are met. Very satisfied, satisfied, neutral, unhappy, and very dissatisfied were the five likert scale questions in the tool. It was divided into two categories: satisfied and very satisfied clients were considered satisfied, while neutral, dissatisfied, and very dissatisfied clients were considered unsatisfied.

### Data collection tools and procedures

Data collection tools were developed from reviewing different literatures and organized to fit this study. Data collection was conducted by using a structured interviewer administered questionnaire. Data collectors were three diploma nurses and one BSc nursing professionals. Patients’ socio-demographic data and their level of satisfaction with health services were collected from each study participants. Participants were interviewed at the exit of the health centers.

### Data quality management and analysis

The questionnaires were first prepared in English and then translated to local language /Amharic/. Data collectors were trained for one day on data collection procedures before the beginning of data collection. The questionnaires were pretested on 5% (27) of the samples in Tita and Kombolcha 03 health centres. Completeness and consistency were checked during data collection and at the end of data collection by the supervisor and investigators.

After checking for completeness and consistency, the data were entered using Epi data version 3.1 and exported to SPSS version 20 software for advanced analysis. Both descriptive and analytical statistical procedures were employed. To identify factors associated with patient satisfaction, logistic regression was conducted. First the association between each independent variable with the outcome variable was assessed separately using bivariable logistic regression. Then those variables with p-value less than or equal to 0.25 were fitted to multivariable logistic regression model to control the possible confounding variables. In multivariable logistic regression, those variables with P–value less than 0.05 were considered as significantly associated with the level of patient-satisfaction. Adjusted Odds Ratio (AOR) at 95% Confidence Interval (CI) was used to indicate the strength of statistical associations of variables.

### Ethical consideration

Ethical clearance was obtained from the ethical review committee of the College of Medicine and Health Sciences, Wollo University. A formal letter of cooperation was given to each Health Centers. After explaining the objective of the study, informed written consent was obtained from the study participants to confirm their willingness for participation. The respondents were notified that they have the right to withdraw at any moment of the interview. Their confidentiality and safety were secured and all the procedures were conducted according to the Helsinki Declaration.

## Results

### Socio-demographic characteristics of the study participants

A total of 537 study participants were included in the study, making 99.4% response rate. Two hundred seventy-one (50.4%) of the participants were males. An estimated 191(35.6%) of the respondents were between the age group of 28 and 37 years. With regards to educational status of respondents, 179(33.3%) of them had college and/or more education level. More than half, 299 (55.6%) of the respondents were married, whereas nearly one-fourth (24.1%) of the respondents were merchants by occupation. About 296 (55.2%) of respondents were visited the health centers more than one times and 382 (71.1%) were paid for their service. Majority of the respondents 243 (45.2%) had a monthly income of more than two thousand Ethiopian Birr (ETB) (Table 1).

**Table 1:** Socio-demographic characteristics of respondents in south Wollo zone health centers, Ethiopia, 2019 (n = 537).

Variable	Category	Number (n = 270)	Percent (%)
Sex			
	Male	271	50.4
	Female	266	49.6
Age			
	18-27yrs	153	28.5
	28-37yrs	191	35.6
	38-47yrs	125	23.3
	47+yrs	68	12.6
Marital status			
	Single	161	30.0
	Married	299	55.6
	Divorced	59	11.1
	Widowed	18	3.3
Educational status			
	Unable to read and write	14	2.6
	Able to read and write	71	13.3
	Grade 1–6	114	21.1
	Grade 7–12	159	29.6
	Diploma and above	179	33.3
Occupation			
	Government employee	111	20.7
	Merchant	129	24.1
	House wife	86	15.9
	Farmer	96	17.8
	Jobless	38	7.0
	Others	77	14.4
Payment status			
	Paying	382	71.1
	Free	155	28.9
Frequency of visit			
	First	241	44.8
	Repeated	296	55.2
Monthly income			
	<1000	91	17.0
	1000–2000	203	37.8
	>2000	243	45.2

### Estimated time taken to arrive at the health centers and resources expended (time and money) in the health centers by respondents

Among the total respondents, 324 (60.4%) of them had a length of 1–2 hours stay in the health centers. Based on the respondents’ rating of the length of stay they had to stay to receive care, the length of stay was reported to be fair for those 234 (43.7%) participants.

Payments at the health centers may be for registration, laboratory investigation, medication, treatment procedures, or any of the combinations. As to the patients’ perception towards the amount of payment requested by the health center for the aforementioned activities/services, 215 (40%) of the respondents rated the payment was fair. Three hundred ten (57.8%) of the respondents reported that the time taken to arrive at the health center was 15-30minutes (Table 2).

**Table 2:** Resources spent and time taken to arrive at the health centers by respondents, South Wollo, Ethiopia, 2019 (n = 537).

Variables	Frequency (%)
Time taken to arrive at the health centers (in minutes)	
< 15	125 (23.3%)
15–30	310 (57.8%)
31–60	102 (18.9%)
Length of stay in the HCs for outpatient service users (in hrs)	
< 1hr	106 (19.6%)
1-2hrs	324 (60.4%)
2-6hrs	107 (20%)
Respondents’ rating of length of stay in the HCs	
Very long	18 (3.3%)
Long	56 (10.4%)
Fair	234 (43.7%)
Short	193 (35.9%)
Very short	36 (6.7%)
Respondents’ rating of the amount of money paid for services in the HCs (n = 382)	
Very cheap	44 (11.4%)
Cheap	87 (22.7%)
Fair	214 (56.0%)
Expensive	37 (9.9%)

### Friendliness of outpatient services and availability of advised medical services or drugs in the health centers

Nearly 467(87%) of respondents reported the outpatient department they visited was convenient to ask questions. Out of all respondents, 527 (98.1%) of them reported that their privacy at the out-patient department was maintained. Five hundred three (93.7%) of respondents declared to have had a good dialogue with outpatient service providers. The scale (politely, neutral, impolitely) was used to assess the degree of politeness of outpatient service providers who served the respondents. Thus, 350 (65.2%) of respondents described outpatient service providers were polite during service provision. Among the total patients interviewed, 296(55.2%) and 294 (54.8%) reported to have got all ordered laboratory tests and drugs from the health centers, respectively. Exactly 483 (90%) and 471 (87.8%) of the respondents wish the health center for their future visit and would like to recommend to visit the health center to their friends or relatives, respectively. The overall patient satisfaction rate of the study was 64.4% with a 95% CI (59.3–67.6%) (Table 3).

**Table 3:** Outpatient service characteristics and perceptions of respondents in south Wollo, Ethiopia, 2019 (n = 537).

Variables	Frequency (%)
The environment was convenient to ask questions	
Yes	467 (87.0%)
No	70 (13.0%)
Patient’s privacy was maintained in the outpatient department	
Yes	527(98.1%)
No	10 (1.9%)
Had good dialogue with outpatient service provider	
Yes	503 (93.7%)
No	34 (6.3%)
Politeness of outpatient service providers	
Polite	350 (65.2%)
Neutral	68 (12.6%)
Impolite	19 (3.7%)
Have got all ordered diagnostic or laboratory tests from the health center	
Yes	296 (55.2%)
No	64 (11.9%)
Not ordered	177 (33.0%)
Have got all ordered drugs from the health center	
Yes	294 (54.8%)
No	243 (45.2%)
Wish the health center for future visit	
Yes	483 (90.0%)
No	54 (10.0%)
Would like to recommend this health center for a friend or relative	
Yes	471 (87.8%)
No	66 (12.2%)
Satisfaction with outpatient services	
Very satisfied	52 (9.6%)
Satisfied	284 (52.8%)
Neutral	93 (17.4%)
Dissatisfied	93 (17.4%)
Very dissatisfied	15 (2.8%)

### Factors associated with patient satisfaction

Binary and multiple logistic regression models were performed to identify factors associated with patients’ satisfaction. Respondents who got all the prescribed drugs from the health centers and have convenient environment to ask questions were more satisfied than their counterparts (AOR = 6.3, 95% CI: 3.2–12.5) & (AOR = 6.97, 95% CI: 3.46–8.55) respectively (Table 4).

**Table 4:** Factors affecting patients’ satisfaction with outpatient services, south Wollo, Ethiopia, 2019 (n = 537).

Variables	Satisfaction status		COR (95% CI)	AOR (95% CI)
	Satisfied	Unsatisfied		
Frequency of visit				
First	146	95	1.38(0.839–2.283)	1.41(0.709–2.810)
Repeated	201	95	1	1
Monthly income				
<1000	60	32	1	1
1000–2000	111	91	1.54(0.749–3.168)	1.56(0.565–4.122)
>2000	175	68	0.72(0.351–1.495)	1.24(.456–3.369)
Time taken to arrive at the health center(in minutes)				
<15	93	32	1	1
15–30	205	111	1.64(.855–3.166)	2.50(0.938–6.685)
31–60	48	48	2.61(1.185–5.754)	2.16(.690–6.803)
Length of stay in the HCs for outpatient service use (in hrs.)				
<1hr	60	46	1	1
1-2hrs	214	109	0.66(.353–1.251)	0.56(.235–1.373)
2-6hrs	72	36	0.65(0.298–1.429)	0.40(.134–1.229)
The environment was convenient to ask questions				
Yes	338	129	20.27(6.880–59.67)	6.97(3.46–8.55)*
No	8	62	1	1
Had good dialogue with outpatient service provider				
Yes	336	167	4.82(1.647–14.156)	0.23(0.028–1.904)
No	10	24	1	1
Politeness of outpatient service providers				
Polite	295	40	1	1
Neutral	41	74	0.0744(0.025–0.225)	0.121(0.031–0.476)**
Impolite	57	30	0.166(0.100–0.246)	0.112(0.091–0.181)***
Wish the health center for future visit				
Yes	338	145	13.39(4.472–40.092)	2.49(0.459–13.604)
No	8	46	1	1
Would like to recommend this health center for a friend or relative				
Yes	298	173	10.95(4.322–27.712)	1.89(0.429–8.414)
No	12	54	1	1
Have got all ordered drugs from the health center				
Yes	249	46	8.09(4.563–14.366)	6.37(3.236–12.550)*
No	97	145	1	1

* = p-value < 0.05

** = p-value< 0.001

*** = p-value < 0.0001.

## Discussion

The aim of this study was to assess the level of patient satisfaction and its associated factors among OPD attending patients at health centers in south Wollo Zone. The overall level of patient satisfaction in this study was 64.4% (95% CI: 59.3–67.6%). This finding is in agreement with the findings of a study carried out at Calabar Teaching Hospital in Nigeria (57.1%), Jimma (57.7%,), Debrebirhan hospital (61.9%), Amhara Region Referral Hospitals (65.9%) [11–14].

Allover patient satisfaction level was lower compared to the reports of the studies conducted in Jimma University Specialized Hospital (77%) and Hawassa University Teaching Hospital (80.1%), India (78%), Egypt (86.2%), Kenya (84%) [5, 6, 15–17]. The difference might be due to the fact that those studies were conducted in specialized teaching and referral hospitals which are equipped very well and have enough diversity of health professionals, better diagnostic facilities, health service infrastructures, and awareness of service providers of different levels that are expected to demonstrate the standard way of patient examination resulting in higher level satisfaction.

On the other hand, this finding was higher than the findings of a study conducted in Eastern Ethiopia (54.1%), Tigray Zonal hospital (43.6%) and Gondar referral hospital (22%) [18–20]. The reason might be one would ordinarily expect a higher level of satisfaction with care received at a tertiary hospitals and hospitals because of the available expertise, technology and sophisticated procedures that can be obtained rather than health centers.

In this study more than half (54.8%) of the respondents have got all prescribed drugs from the health centers pharmacy. Similar study conducted in Jimma University Specialized Hospital (70%), Amhara regional hospitals (66.7%), Tigray Zonal hospital (61%), Jimma hospital (1/3rd of total clients) of the patients didn’t get all or some of prescribed drugs from hospitals pharmacy and lack of drugs and supplies were their major problems, which were in contrast to the findings of this study [5, 12, 14, 20]. On the other hand, patient satisfaction for the availability of prescribed drugs from the health institutions pharmacy was high in studies conducted in India (75%), Adama (65%) and Wolayita sodo hospital (64.3%) as compared to this result [17]. This may be due to the reason that hospitals are well organized in all health facility services than health centers.

Patients’ satisfaction with politeness of health service provider’s was higher at the health centers. This may have resulted from the lower demand for general practice care at the health center when compared with the daily influx of patients that seek such services at the teaching hospitals. High patient turnover, occupational stress and stringent work targets could affect the communication and interpersonal relationship between patients and providers. There is a link between communication capabilities of clinicians and patient satisfaction. Ineffective communication like unfriendliness and discourtesy by doctors, insufficient explanations on diagnosis and management protocol have been implicated in the dissatisfaction of patients with health care [21, 22].

Patients who did not claim the existence of a convenient environment to ask questions and patients who did not have a good dialogue with outpatient health service providers were less satisfied. A study carried out in health centers in central Ethiopia also revealed that good dialogue and non-verbal communications to be predictors of high degree of patients’ satisfaction [23]. This is also supported by a study conducted in United Arab Emirates at public hospitals which identified perceived welcoming approach of service providers as a significant determinant of patient satisfaction [24]. A study conducted in South Africa revealed lack of communication and relevant messages to patients were identified as an important issue impacting on quality thus affecting client satisfaction [25].

## Limitations

There are no accessible similar studies done at the health centers level. This leads not to compare the findings with the similar institution services. The finding of this study might be subjected to social desirability bias because the respondents were interviewed in the health centers compound.

## Conclusions

Patients attending health centers are messenger of spreading good images of the health centers and therefore patients’ satisfaction is equally important for health centers management to improve the health care quality. The overall patient satisfaction of this study was low. Environmental convenience to ask questions, politeness of service providers and availability of ordered drugs were statistically significant factors for patient satisfaction. Management bodies need to give attention to the physical cleanness of the health centers, patient safety and privacy, and fulfilment of essential drugs to improve their patients and/or customers’ satisfaction from their service provision.
