# Expression of Concern: Level of satisfaction and associated factors among patients attending outpatient departments of south Wollo health facilities, Ethiopia

**DOI:** 10.1371/journal.pgph.0004902

**Published:** 2025-07-07

**Authors:** The PLOS Global Public Health Editors

The *PLOS Global Public Health* Editors issue an Expression of Concern to alert readers that this article [1] was identified as one of a series of submissions for which we have concerns about authorship and adherence to the journal’s first and second publication criteria. We regret that these issues were not addressed prior to the article’s publication.

Contrary to the declaration in the Data Availability statement, the original raw data files supporting the article’s results were not provided with the article, and the authors have not provided these data following editorial request. As the original raw data files supporting the article’s results have not been made available, this article [1] does not comply with the PLOS Data Availability policy.
